# Effects of Inorganic and Organic Pollutants on the Biomarkers’ Response of *Cerastoderma edule* under Temperature Scenarios

**DOI:** 10.3390/antiox12091756

**Published:** 2023-09-13

**Authors:** Andreia F. Mesquita, Fernando J. M. Gonçalves, Ana M. M. Gonçalves

**Affiliations:** 1 Department of Biology and CESAM—Centre for Environmental and Marine Studies, University of Aveiro, 3810-193 Aveiro, Portugal; filipamesquita@ua.pt (A.F.M.); fjmg@ua.pt (F.J.M.G.); 2 University of Coimbra, MARE—Marine and Environmental Sciences Centre/ARNET—Aquatic Research Network, Department of Life Sciences, Calçada Martim de Freitas, 3000-456 Coimbra, Portugal

**Keywords:** chemical pollution, pesticides, metals, climate change, marine bivalves, biochemical biomarkers

## Abstract

Currently, there is increased chemical pollution, and climate change is a major concern to scientific, political and social communities globally. Marine systems are very susceptible to changes, and considering the ecological and economic roles of bivalve species, like *Cerastoderma edule*, studies evaluating the effects of both stressors are of great importance. This study intends to (a) determine the toxicity of copper (Cu) and oxyfluorfen at the lethal level, considering the temperature; (b) assess the changes in the antioxidant defence enzymes as a consequence of the simultaneous exposure to chemical and warming pressures; and (c) determine if lipid peroxidation (LPO) and neurotoxic effects occur after the exposure to chemical and temperature stressors. *C. edule* was exposed to Cu and oxyfluorfen at different temperatures (15 °C, 20 °C and 25 °C) for 96 h. The ecotoxicological results reveal a higher tolerance of *C. edule* to oxyfluorfen than to Cu, regardless of the temperature. The antioxidant defence system revealed efficiency in fighting the chemicals’ action, with no significant changes in the thiobarbituric reactive species (TBARS) levels to 15 °C and 20 °C. However, a significant inhibition of acetylcholinesterase (AChE) was observed on the organisms exposed to oxyfluorfen at 20 °C, as well as a decreasing trend on the ones exposed to Cu at this temperature. Moreover, the catalase (CAT) showed a significant increase in the organisms exposed to the two highest concentrations of Cu at 15 °C and in the ones exposed to the highest concentration of oxyfluorfen at 20 °C. Looking at the temperature as a single stressor, the organisms exposed to 25 °C revealed a significant increase in the TBARS level, suggesting potential LPO and explaining the great mortality at this condition.

## 1. Introduction

The exponential increase in the human population, which is predicted to reach about 10 billion people by 2050 [1,2], emphasises the increasing food needs and, consequently, the intensive use of pesticides. Therefore, in the past three decades, pesticide consumption increased by about 1 million tonnes [3]. Pesticides are used to improve the yield and quality of harvests through pest control (e.g., insects, fungi, weeds, and rodents) [4], but among the 4.07 million tonnes of pesticides applied worldwide [5], only 1% reaches the target organisms, with the remaining 99% inputted on soils and water bodies [6], with effects on the ecosystems and their communities. Pollutants may be classified as organic or inorganic, with the organic contaminants being reported as the most powerful pesticides, as they are designed to block key processes and consequently kill the organisms [7]. Copper (Cu) is used in several inorganic formulations and is applied in agricultural practices as a pesticide and fertiliser [8]. Cu is an essential element with key roles in several biological processes, but at high concentrations, it becomes toxic [9,10,11,12,13]. Regardless of its toxicity, concentrations from 10 mg L^−1^ in urban aquatic bodies to 100 mg L^−1^ in aquatic systems surrounding certain places have been reported in the environment [14]. On the one side, oxyfluorfen is an organic pesticide, and is one of the main herbicides used worldwide [15]; it is reported as an alternative to glyphosate-resistant weeds, and consequently, its application has increased [16]. However, toxic effects, such as neurotoxic, carcinogenic and potentially mutagenic effects, and at the last level, lethality, have been reported in non-target species [17,18,19,20]. Furthermore, high concentrations are reported in the environment, reaching 23.6 mg L^−1^ in the Nile River (Egypt) [21]. 

Climate change, paired with chemical pollution, is currently a major concern globally, and recent studies predict an increase of about 4 °C/5 °C in the Earth’s temperature by the end of the 21st century [22,23]. In addition to climate change and global warming being shown to affect crops and food production globally [24], these phenomena comprise other consequences to aquatic ecosystems and their organisms. 

The temperature increase has been reported as a great influencer of the life cycle of marine organisms, since aquatic systems can be affected even by slight temperature changes [25]. The authors of [26] predict that a temperature increase leads to a moderate to high risk of impact on marine organisms, with the predicted risk of impact on bivalve species being high to very high. In the past decade, some studies have been dedicated to understanding the temperature impact on bivalve species at the lethal level [27], but also at the sub-lethal level, namely considering the effect on growth and reproduction [28], metabolism and oxidative stress [29,30]. However, the few studies that are focused on the effect of temperature on pollutants’ behaviour and toxicity, as well as the changes in the toxicity pattern and on the organisms’ response, are relatively recent [31,32,33,34]. 

Bivalve species have important roles in the balance of marine and estuarine systems because they act as links between primary producers and consumers (e.g., crustaceans, fish and wading birds); due to their high ability to filtrate and accumulate particulates, they also contribute to water cleaning and purification, and participate in carbon and nutrient cycles [35]. Furthermore, the marine bivalve *C. edule* is greatly appreciated as a food source by the human population, which confers it an interesting economic value [36,37]. Thus, considering the physiological characteristics of *C. edule*, namely their long life span between 6 and 10 years; their reproductive maturity age (1–2 years); sessile lifestyle; ease of collecting, handling and maintenance in the laboratory; their great ability to filter and accumulate particulates, including pollutants; and their sensitivity to chemicals, these organisms have been reported as good bioindicators and are largely used in ecotoxicological and monitoring studies [8,11,27,36,37,38,39,40,41,42]. *C. edule* are widely distributed worldwide, inhabiting waters between 15 °C and 20 °C [27].

According to the best of our knowledge, this is the first work evaluating the impact of temperature on the toxicity of Cu and oxyfluorfen on the marine bivalve species *Cerastoderma edule*, considering lethal and sub-lethal endpoints. So, this study intends to (a) determine the lethal concentrations (LCs) of Cu and oxyfluorfen at different temperatures, considering the species’ distribution (15 °C and 20 °C) to *C. edule*; (b) assess changes induced by Cu and oxyfluorfen depending on the temperature and on the antioxidant defence response, namely evaluating alterations on the enzymatic activity of Glutathione S-transferase (GST), Glutathione Reductase (GR), Glutathione Peroxidase (GPx) and Catalase (CAT); (c) predict the occurrence of lipid peroxidation (LPO), considering the measurement of thiobarbituric reactive species (TBARS) as a consequence of exposure to chemical and temperature stressors; and d) determine if temperature influences the neurotoxic effects of both chemicals through the determination of acetylcholinesterase (AChE) activity. 

## 2. Materials and Methods

### 2.1. Sampling Site and Organisms’ Acclimation

*Cerastoderma edule* was collected in April 2022 in Ria de Aveiro (Aveiro, Portugal: N 40°38.52060′; W 8°44.12840′), with a total of 650 organisms from the field. Then, the organisms were immediately carried to the laboratory in cold boxes with field water. In the laboratory, organisms were transferred to glass aquariums, with previously filtered seawater (in a total volume of 10 L of water) at a salinity of 20 PSU, and measured through a multiparameter WTW Multi 3430 SET F (WTW—London, UK). In the acclimation period, 600 organisms were divided in the aquariums, each with 100 organisms; these were kept there for 2 days with no food and with constant aeration at 20 °C. After this time, the organisms were fed daily, ad libitum, with a frozen commercial mixture of microalgae and rotifers (from Ocean Nutrition^®^—Dartmouth, NS, Canada), and the water was renewed every 48 h.

When the bivalves arrived at the laboratory, 10 organisms were randomly selected, measured, weighed and dissected to assess biochemical responses. After acclimation, the other 10 organisms were randomly selected and submitted to the same procedure. Then, 580 organisms were randomly selected for the bioassays (please see Section 2.3).

### 2.2. Chemicals 

Oxyfluorfen (CAS: 42874-03-3, PESTANAL^®^ analytical standard) was acquired from Sigma-Aldrich (Algés, Portugal) and copper (II) sulphate pentahydrate (CAS: 7758-99-8, EMSURE® ACS, ISO, Reag. Ph Eur) was acquired from Merck (Algés, Portugal). A stock solution of oxyfluorfen was prepared using acetone as the solvent, and a stock solution of copper (II) sulphate pentahydrate was prepared in previously filtered seawater at 20 PSU of salinity. The maximum concentration of acetone in the medium was 0.008%, which was lower than the maximum recommended by OECD guidelines. Only chemicals of analytical grade were applied in the bioassays and biochemical analyses.

### 2.3. Bioassays

Considering the variation between the nominal and measured chemical concentrations, the nominal concentrations will be considered throughout the whole work (please see Section 3.1 for more details).

The organisms previously submitted to the acclimation time were exposed to distinct bioassays, for 96 h, according to the following conditions: temperatures of 15 °C, 20 °C and 25 °C, chemicals including Cu (from 81.25 µg L^−1^ to 605.58 µg L^−1^) or oxyfluorfen (from 1.88 mg L^−1^ to 21.36 mg L^−1^), plus a negative control and a solvent control for the experiments with oxyfluorfen. The concentration range of each chemical was defined by considering the existent bibliography about the concentrations found in the environment as well as the lethal concentrations determined for the other bivalve species according to a factorial ratio of 1.5 X.

Water samples were collected at the beginning and the end of the bioassays and preserved for later Cu and oxyfluorfen quantification.

Bioassays were performed in plastic and glass vessels to Cu and oxyfluorfen, respectively, according to the OECD guidelines, which recommend plastic vessels for inorganic chemicals, such as Cu, and glass vessels for organic chemicals, such as oxyfluorfen. Each treatment was composed of 4 replicates, each one with 3 organisms per vessel, in a volume of 300 mL of medium, with constant aeration. Organisms were fed daily with the frozen commercial mixture ad libitum, and the medium was renewed every 48 h. 

At the end of the bioassay, 6 survival organisms per treatment (considering only the treatments with a maximum of 30% mortality) were randomly selected, measured, weighed, dissected and stored at −80 °C for further biochemical analyses (Section 2.4). Moreover, the other 3 survival organisms were also selected, measured, weighed, dissected and stored at −80 °C for the quantification of the chemical on the organisms’ tissues (Section 2.7).

### 2.4. Biochemical Analyses

Chemicals and temperature effects on the protein concentration and antioxidant enzymatic activity were assessed in the soft tissue to determine oxidative stress enzyme activity (CAT, tGPx, GR and GST), as well as the lipid peroxidation through TBARS measurement, and neurotoxic effect via the determination of the AChE activity via spectrometry. 

Homogenisation phosphate buffer (50 mM, pH 7.0 with 0.1% Triton X-100) allowed for soft tissue homogenisation using a homogeniser Ystral D-79282 (Ballrechten-Dottingen, Germany). Then, the homogenised tissue was centrifuged at 15,000× *g* for 10 min, and the supernatants were collected and stored at −80 °C until posterior biochemical analyses. Protein, enzymatic activity and lipid peroxidation were determined in 6 organisms per treatment and compared to the protein content of the corresponding sample as nanomoles of substrate hydrolysed per minute and mg of protein, unless otherwise indicated.

All biomarker determinations were performed using a microplate reader, the Biotek Synergy H1Multimode Reader (Winooski, VT, USA).

#### 2.4.1. Total Protein Concentration

The total protein concentration of each sample was quantified in quadruplicate at 595 nm according to the Bradford method [43], and adapted to microplates. Samples of protein concentration were achieved by comparison with the standard γ-bovine globulin.

#### 2.4.2. Catalase (CAT)

CAT activity was determined in triplicate according to [44], and adapted to microplates as described by [45]. Briefly, phosphate buffer (0.05 M, pH 7.0) and hydrogen peroxide (H_2_O_2_; 0.010 M) were added to the supernatant, and the decrease in absorbance due to the degradation of H_2_O_2_ was measured in UV-transparent microplates (flat-bottom microplates, Greiner Bio-One GmbH (Frickenhausen, Germany) at 240 nm during 3 min with intervals of 10 s.

#### 2.4.3. Total Glutathione Peroxidase (tGPx)

tGPx (EC 1.11.1.9) activity was assessed in triplicate according to [46], using a phosphate buffer (100 mM, pH 7.0). NADPH oxidation was followed for 5 min, at 340 nm, using cumene hydroperoxide (0.7 mM) as substrate.

#### 2.4.4. Glutathione Reductase (GR)

GR (EC 1.8.1.7) activity was quantified in triplicate according to the method of [47], using a phosphate buffer (200 mM) with EDTA (2 mM) and pH = 7.0. GR with a part on the NADPH oxidation was followed by 5 min at a wavelength of 340 nm. 

#### 2.4.5. Glutathione S-Transferases (GSTs)

GST (EC 2.5.1.18) activity was assessed in triplicate according to [48] using a phosphate buffer (0.1 M) with pH = 6.5. Thioester formation resulted from the conjugation of substrate 1-chloro-2, 4-dinitrobenzene (CDNB) with the reduced glutathione (GSH) via GST action and was monitored for 5 min via the increase in absorbance at 340 nm. 

#### 2.4.6. Thiobarbituric Acid Reactive Species (TBARS)

TBARS were measured, in duplicate, according to [49]. In a single read at 535 nm, the reaction of lipid peroxidation by-products (such as malondialdehyde, MDA) with 2-thiobarbituric acid was evaluated, and results are expressed as nmol of MDA equivalents per mg of sample protein.

#### 2.4.7. Acetylcholinesterase (AChE)

AChE activity was determined in quadruplicate according to the method described in [50], and adapted to microplates using the method described in [51]. Briefly, a reaction solution was prepared, containing phosphate buffer (0.1 M; pH = 7.2), acetylcholine (0.075 M) and DTNB (10 mM). AChE disintegrated the acetylcholine into ticholine and acetate, and the complexation between ticholine and DTNB resulted in a yellow by-product, monitored every 5 min at 412 nm. 

### 2.5. Determination of the Integrated Biomarker Response (IBR)

The IBR version 2 (IBRv2) was defined using the results of the biomarker analyses to provide a full overview of the stress response of *C. edule* at each treatment. The IBRv2 was calculated following [52], using Microsoft Office Excel 2013 software. 

For each biomarker, the function Yi = log (Xi/X0), where Xi is the individual data points and X0 is the control mean value. Further, the data were standardised as follows: Zi = (Yi − I)/r, where I indicates the general mean, and r indicated the standard deviation. Then, the biomarker deviation index (A) was calculated using the formula A = Zi − Z0, where Z0 means the Z value for the control treatment. The deviation index of each biomarker (A) regarding the control is displayed in a star plot. The IBRv2 values for each biomarker to each condition were achieved through the sum of the absolute A values (IBRv2 = ∑ |A|).

### 2.6. Data Treatment and Statistical Analyses

Significant differences between each treatment and control, based on the protein concentration, enzymatic activity and TBARS amount, were evaluated through a one-way variance analysis (ANOVA), followed by Dunnett’s multiple comparisons test. ANOVA assumptions were previously checked, namely normality (Shapiro–Wilk test) and homoscedasticity (Levenes’ test). When ANOVA assumptions were not fulfilled, the non-parametric Kruskal–Wallis test, followed by Dunn’s test, was used as an alternative. 

A significance level of 0.05 was used for all statistical tests.

### 2.7. Chemicals Quantification

The water samples for oxyfluorfen quantification were concentrated via solid phase extraction (SPE) according to [53]. Moreover, oxyfluorfen was quantified on tissues according to the method described in [54]. Then, oxyfluorfen concentrations on water and tissue were achieved via gas chromatography equipped with an electron capture detector (GC-ECD), namely Agilent 7890 B (Wilmington, DE, USA); the procedure was based on the method described in [55]. The detection limit (LOD) was 3.53 μg L^−1^ and the quantification limit (LOQ) was 10.68 μg L^−1^. 

Regarding Cu quantification, the preparation of water and tissue samples was performed according to the procedure described in [56]. Briefly, water samples were acidified with supra pure nitric acid Pro Analysis MERCK^®^ (65%) (Algés, Portugal) to a pH below 2 and stored at 4 °C until the chemical analyses, and the tissues were dried, weighed and digested with nitric acid; after, the organic matter was destroyed with hydrogen peroxide. Cu concentrations were measured via flame atomic absorption spectrophotometry (ICE3000 Series AA Spectrometer—Thermo Scientific—Winsford Cheshire, UK). The LOD was 6.60 μg L^−1^ and the LOQ was 19.99 μg L^−1^. 

The measured concentrations of each pesticide are presented in Table 1.

## 3. Results and Discussion

### 3.1. Chemical Analyses of Medium and Tissues Used in Biochemical Analysis

The concentrations of Cu and oxyfluorfen were measured in the water and the organisms’ tissues and they are described in Table 1. Considering the variation between the nominal and measured concentrations from 96.4% to 99.30% for Cu and from 85.9% to 102.6% for oxyfluorfen, the nominal concentrations will be considered in all of the work. 

The chemical data show that the organisms exposed at 20 °C had higher tissue concentrations than those exposed at 15 °C, even though the exposure concentrations in water were the same. These results are corroborated by those of other studies, such as in [57], which associated a higher bioaccumulation of contaminants to the highest temperatures, as well as in [33], which reported a higher bioaccumulation of neodymium in *Mytilus galloprovincialis* when exposed at 21 °C than when exposed to 17 °C, and a higher bioaccumulation of lead when *M. galloprovincialis* was exposed to 22 °C than when exposed to 17 °C [58]. The higher bioaccumulation at the highest temperature can be associated with the higher mobility and transport of chemicals at the highest temperatures, as reported in [59,60].

### 3.2. Lethal Effects

The results shown in this work were observed after 96 h of exposure, considering the ability to maintain this species under laboratory conditions. Different results could be observed in a longer exposure period, with different lethal concentrations and different biochemical responses, depending on the organisms’ acclimatation abilities. However, considering this particular species, longer studies must be conducted in situ, since after two weeks in laboratory conditions, the organisms start to die naturally, invalidating the possibility of evaluating the real effect of any stressor.

The ecotoxicological results show that Cu is more toxic to *C. edule* than oxyfluorfen regardless of the temperature of exposure (LC_50_ (15 °C) = 0.229 (0.199–0.265) mg L^−1^; LC_50_ (20 °C) = 0.209 (0.176–0.248) mg L^−1^ of Cu; LC_50_ (15 °C) = 22.735 (16.324–48.510) mg L^−1^; and LC_50_ (20 °C) = 18.052 (11.238–44.868) mg L^−1^ of oxyfluorfen) (Table 2). Previous studies conducted by our research team observed this pattern of a higher sensitivity of bivalve species to Cu than to other herbicides, namely *C. edule* and *Scrobicularia plana* exposed to CuSO_4_ (LC_50_ = 0.818 (eq. 0.205 Cu) mg L^−1^ and 2.563 (eq. 0.641 Cu) mg L^−1^, respectively) [8] and to the herbicide Primextra Gold TZ (LC_50_ = 28.784 mg L^−1^ and 13.26 mg L^−1^, respectively) [41]. The authors of [61] also reported high LC_50_ values in organic chemicals on S. plana, namely S-metolachlor (LC_50_ = 40.702 mg L^−1^) and terbuthylazine (LC_50_ = 118.590 mg L^−1^). Moreover, organic pesticides were revealed as being more toxic to different species than metal contaminants. The authors of [62] determined LC_50_ = 3.7 mg L^−1^ for copper (I) chloride (eq. 2.37 mg L^−1^ Cu) on *Ruditapes decussatus*, whereas the authors of [21] reported LC_50_ = 5.96 mg L^−1^ for oxyfluorfen on *Biomphalaria alexandrina* and the authors of [63] reported LC_50_ = 7.77 g L^−1^ for the insecticide Calypso 480 SC on *Mythilus galloprovincialis*. The higher sensitivity of bivalve species to Cu than to organic pesticides, such as oxyfluorfen, can be explained by the pesticide action mode, which is designed to act essentially on vegetal cells, while Cu is non-selective, and regardless of its essential role in the biological functions of organisms at trace levels, it becomes toxic at high levels. Additionally, by comparing the oxyfluorfen lethality between *C. edule* and other studies with fish species, the chemical showed to be more toxic to fish species, such as *Clarias gariepinus* (LC_50_ = 11.698 mg L^−1^), *Danio rerio* (LC_50_ = 6.654 mg L^−1^) and *Oreochromis niloticus* (LC_50_ = 3 mg L^−1^) [17,64,65]. 

The temperature increase also exhibited a negative effect on the cockles’ survival (Table 2), which is in concordance with the results from [29] on *Crassostrea virginica* and *Mercenaria mercenaria*, with the results in [58] on *M. galloprovincialis* and the results in [27] on *C. edule* and *S. plana*, respectively. 

The determination of LC values was not possible for *C. edule* exposed at 25 °C, because at this temperature, the mortality in the control treatment achieved 20%, which was in concordance with the results obtained in [27], and it was higher than the tolerance of 10%, invalidating the bioassay. 

According to the weather data, in the past 7 years, the temperature average in Ria de Aveiro was between 12 °C and 17 °C in the coldest months and between 14 °C and 22 °C in the warmest months [66]. However, the observed results must represent a great concern, because the prediction models suggest that an increase of 8.8 °C in the air temperature translates into an increase of 3 °C to 4 °C in the water temperature in Ria de Aveiro [67]. According to this, the increase of 4 °C to 5 °C in the air temperature by the year 2100, predicted by the IPCC, can translate into an increase of 2 °C to 3 °C in the water temperature in Ria de Aveiro, and the temperature average can increase to 19 °C/20 °C in the coldest months or to 24 °C/25 °C in the warmest months. 

### 3.3. Biochemical Responses

**Figure 1:**
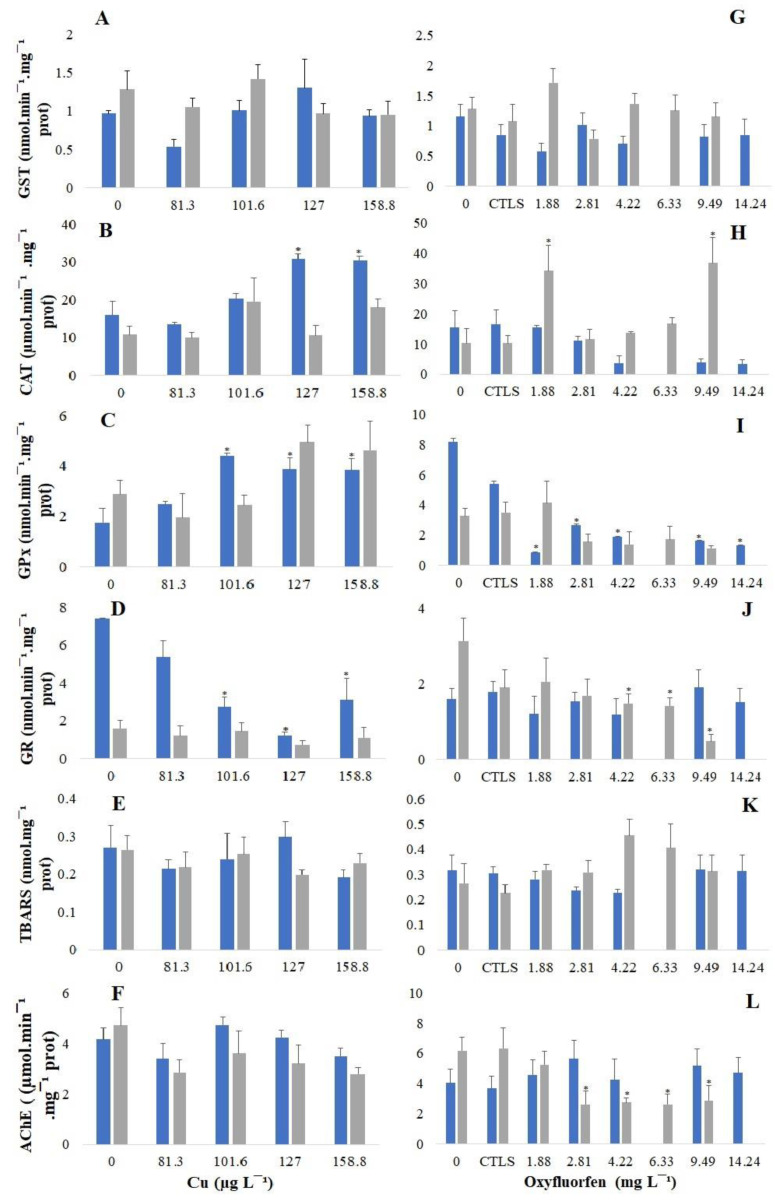
Biomarkers’ response of *C. edule* exposed to Cu and oxyfluorfen at different temperatures (15 °C—blue bars; 20 °C—grey bars) after 96 h of exposure. Activity of the enzymes GST (**A**,**G**), CAT (**B**,**H**), GPx (**C**,**I**), GR (**D**,**J**), TBARS (**E**,**K**) and AChE (**F**,**L**). The standard error is represented by the error bars, and treatments with a statistically significant difference regarding the control (0) are indicated with an asterisk. Note that different scales were used according to the enzyme activity measurement.

Marine bivalves have been described as good bioindicators of metals and pesticide presence [68]. Highlighting their potential as bioindicators, the present work demonstrated that biochemical responses are dependent on the chemical and temperature of exposure (Figure 1). The data show that the GST (Figure 1A and G) was the less responsive among the antioxidant defence enzymes; the same was observed by previous studies with *C. edule* [11,45,69]. The opposite was observed for CAT (Figure 1B and H), GPx (Figure 1C and I) and GR (Figure 1D and J). That seems to be responsive antioxidant enzymes, with different patterns for each contaminant. Regarding the CAT, it exhibited a clear trend in the organisms exposed to 15 °C, increasing significantly in the organisms exposed to Cu. Similar results were observed in other marine bivalve species, such as *R. decussatus* [62], *M. galloprovincialis* and *Perna perna* [70]. Moreover, organisms exposed to 20 °C exhibited a slight increase in the CAT when exposed to Cu and oxyfluorfen, with this increase being significant at 1.88 mg L^−1^ and 9.49 mg L^−1^ of oxyfluorfen. CAT acts in the cell protection against the hydrogen peroxide action, converting it into water and oxygen [71,72]; it is one of the enzymes responsible for the first step of the antioxidant defence system to deal with the overproduction of oxygen reactive species (ROS) during the pollutants’ biotransformation processes [71,73,74,75]. Some works have observed that oxyfluorfen induces CAT activity in mollusc species, such as *B. alexandrina* [21], and fish species, namely *O. niloticus* [20] and *C. gariepinus* [64]. The authors of [76] also reported an increase in CAT activity when *Donax incarnatus* was exposed to the pesticide monocrotophos. However, the biomarker responses depend on the chemical tested, the species and the abiotic conditions, and the organisms exposed to oxyfluorfen at 15 °C exhibit a decrease in CAT activity, but not significantly, comparably. The authors of [77] also reported a decrease in this enzyme activity on *M. galloprovincialis* when exposed to REX (a glyphosate formulation). GPx acts on the ROS’ metabolism by the reductions of H_2_O_2_ to water and organic peroxides to stable alcohols [78,79]. So, often, the GPx increases with contamination [11,78], suggesting the need to maintain protection against oxidative stress enhanced by contaminants [79]. The results of this work presented a clear increase trend with the exposure to Cu, with this increase being significant in the organisms exposed to the three highest concentrations at 15 °C; a similar response was observed in previous studies with *C. edule* [11], as well as on *R. decussatus* [62] and *Lymnaea natalensis* [80], and the same was found for *D. incarnatus* exposed to monocrotophos [76]. However, the opposite (a decrease) can also occur, as reported by [42], when *C. edule* was exposed to wildfire ash extract (a complex matrix richness in PAHs), which is similar to the trend observed with the *C. edule* exposure to the organic contaminant oxyfluorfen. Moreover, this decrease was significant in the organisms exposed to all concentrations of oxyfluorfen at 15 °C. The GPx decrease suggests a decrease in the defence systems and the organisms’ inability to fight the oxidative toxicity caused by the contaminants [79]. On the other side, the GR showed a clear decreasing trend for all the conditions of temperatures and chemicals, except for the organisms exposed to oxyfluorfen at 15 °C; also, significant inhibition was observed for the organisms exposed to Cu at 15 °C and for the ones exposed to oxyfluorfen at 20 °C. In an overview, the antioxidant defence system seemed to be effective, as there were no observed changes in the TBARS levels, suggesting no occurrence of lipid peroxidation, which is in concordance with that observed for *C. edule* and *S. plana* when exposed to Cu [11] and for *M. galloproviancialis* when exposed to REX [77]. Despite the efficacy of the antioxidant defence system, a slight decreasing trend was observed for AChE when the organisms were exposed to Cu at 20 °C, and the inhibition was significant to the ones exposed to oxyfluorfen at this temperature, suggesting potential neurotoxic damage caused by the exposure to both chemicals at 20 °C. The inhibition of AChE activity was also reported because of oxyfluorfen exposure on the fish *C. gariepinus* [64], as well as for bivalves exposed to other organic contaminants, from pharmaceuticals to different pesticides. The authors of [81] stated that AChE inhibition with the exposure to carbamazepine for *Ruditapes philippinarum* corroborated the results reported by [82] for *P. viridis*. Moreover, pesticides such as glyphosate or monocrotophos have also been reported as responsible for the AChE inhibition of *P. perna* [83], *M. galloprovincialis* [77] and *Flexopecten glaber* [84]. 

**Figure 2:**
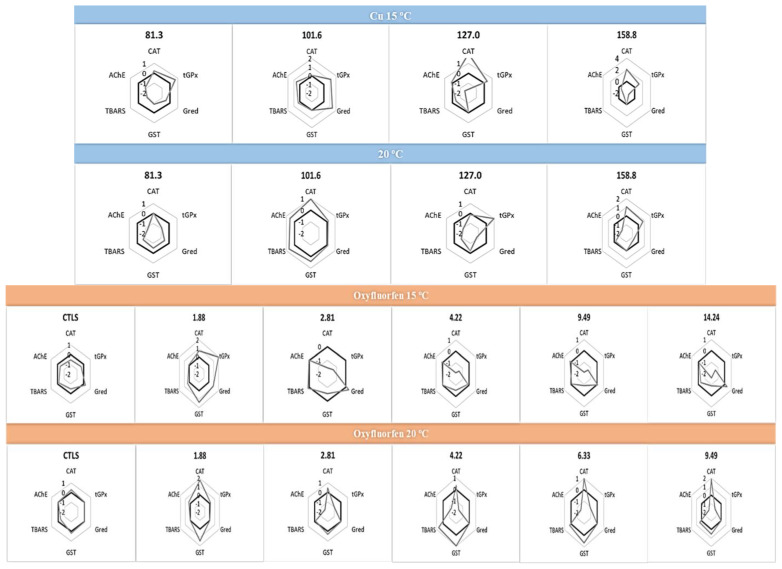
Integrated biomarker response (IBRv2) values of *C. edule* after exposure to Cu and oxyfluorfen at 15 °C and 20 °C, after 96 h of exposure at different treatments, is illustrated relatively to the control organisms (black line; 0). Biomarker induction is indicated for values above 0, whereas inhibition is indicated for values below 0.

The results from IBRv2 (Figure 2) give an integrated view of the biomarkers’ response, along with the different concentrations of each chemical to the different temperatures. Briefly, Cu seems to induce CAT and GPx and seems to inhibit GR at both temperatures, considering the two highest concentrations. Additionally, AChE exhibits a reduction in activity at 20 °C with the two highest Cu concentrations. Furthermore, at 15 °C, Cu causes the same pattern in the biomarkers on the organisms exposed to the two highest concentrations, intensifying along them. Considering these organisms exposed to oxyfluorfen, the biomarkers show different patterns depending on the temperature of exposure; however, the pattern of each temperature was intensified along the concentrations, particularly the decreases in the CAT, GST and tGPx along the three highest concentrations at 15 °C, as well as the increases in the CAT, GST and TBARS and the decreases in the tGPx and AChE along the four highest concentrations when the organisms were exposed to 20 °C. Considering this evidence, IBR v2 seems to be a good tool to predict the chemical effects on the biochemical responses of the marine bivalve *C. edule*, particularly considering the pattern observed for Cu (15 °C) and oxyfluorfen (both temperatures). Further, it highlights the need to consider the temperature as an important factor, as the response pattern to the chemical action is dependent on the temperature. Recent efforts have been made by the scientific community to understand the temperature’s impact on the chemical’s toxicity, but the studies are still few. Despite this, high temperatures have been reported as stimulators of the chemical toxicity to mollusc species, such as *M. galloprovincialis* [58] and *Lymneae* sp. [85], which should be due to the increase in the pesticides’ bioaccumulation [57], mobility and transport with the temperature rise [59,60].

**Figure 3:**
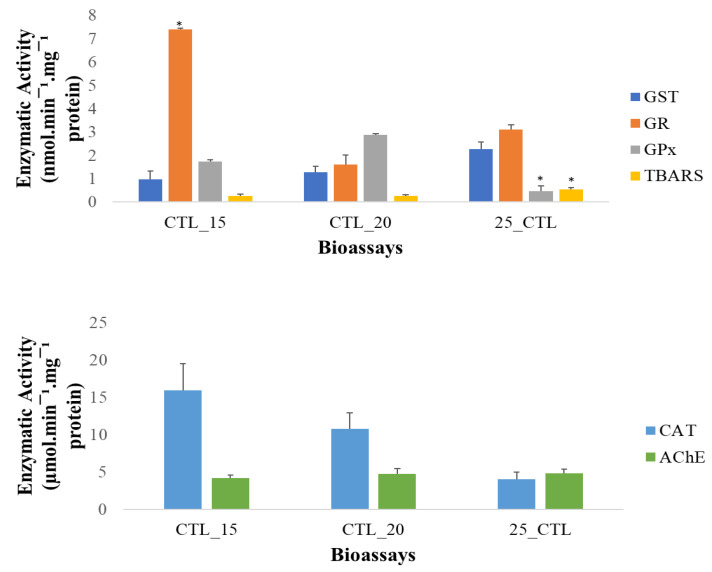
Biomarkers’ response of *C. edule* exposed to the control treatment of each temperature (15 °C, 20 °C and 25 °C) after 96 h of exposure: activity of the enzymes GST (dark blue), CAT (light blue), GPx (grey), GR (orange), TBARS (yellow) and AChE (green). The standard error is represented by the error bars, and biomarkers with a statistically significant difference regarding the remaining control conditions are indicated asterisks.

Biochemical analyses were not performed at 25 °C; as cited above, the mortality for the control treatment achieved 20%, invalidating the bioassay. However, this study also explored the changes in the biomarker responses, considering only the variable temperature (Figure 3).

Considering all the control treatments, at the different temperatures, no significant differences were observed between 15 °C and 20 °C, except for the activity of the GR, but with no consequences on the TBARS or the other enzymes’ activities. However, in the organisms exposed to 25 °C, a significant inhibition of GPx was observed, with no significant changes in the remaining antioxidant defence enzymes, but they were accompanied by a not significant but clear trend of a decrease in the CAT activity, resulting in a significant increase in the TBARS level. Concordantly, the temperature increase has been associated with oxidative stress due to the unbalance between the ROS generation and elimination [86], which suggests the occurrence of lipid peroxidation, potential damage to the lipids and a possible cause of the organisms’ lethality at this temperature even without chemical stressors.

Underlining the great ecological value of *C. edule*, which acts as a link between primary producers and top consumers (e.g., shrimps and wading birds), as well as its role in filtration and water purification [11] and in carbon and nutrient cycles [87], the impacts from stressors, such as chemicals and temperature, observed on these organisms will affect their abundance, and consequently, the population, with impacts on the community structure and function, causing potential disturbances in the habitat stability and cohabitant species. Furthermore, considering the economic value of *C. edule*, its high lethality caused by the exposure to stressors may represent a financial loss for those who sell it, and in the last instance, the biochemical effects observed may represent a risk to human health [54]. 

## 4. Conclusions

The present study described a higher sensitivity of the bivalve species *C. edule* to Cu than to the organic pesticide oxyfluorfen (LC50 (15 °C) = 0.229 (0.199–0.265) mg L^−1^; LC50 (20 °C) = 0.209 (0.176–0.248) mg L^−1^ of Cu; LC50 (15 °C) = 22.735 (16.324–48.510) mg L^−1^; and LC50 (20 °C) = 18.052 (11.238–44.868) mg L^−1^ of oxyfluorfen). Moreover, the temperature increase also enhances *C. edule* mortality, not allowing for the assessment of the chemicals’ lethal concentrations to these organisms at 25 °C, as only the temperature stressor is harmful to this species.

The chemical and temperature effects were not only observed at the lethal level, but also at the biochemical level, with changes in the antioxidant defence response depending on the chemical and temperature. This results in the inhibition of AChE activity at the highest concentrations of oxyfluorfen in the organisms exposed to 20 °C. Moreover, when evaluating only the temperature effect, the significant decrease in the GPx activity in the organisms exposed to 25 °C was also denoted, which was joined with the clear (but no significant) decrease in the CAT activity caused the TBARS increase at this temperature, suggesting lipid peroxidation occurrence. Considering the ecological and economic importance of *C. edule*, these results must be reason for concern. Moreover, *C. edule* is reported by different authors as being highly tolerant to environmental changes, such as salinity or temperature, and considering the effects observed on this tolerant species, it is important to understand the changes’ effects on more sensitive species in the future. So, more studies evaluating the temperature effects on the pollutant’s toxicity should be conducted, and diagnosis and protection measures should be taken.

Overall, this work highlights the temperature’s role in the chemicals’ toxicity, assuming a critical importance on lethal and sub-lethal effects. According to our knowledge, this is the first study establishing the comparative effects of oxyfluorfen and copper on marine bivalve species and considering the role of temperature in the toxicity of both contaminants on these organisms. Additionally, the current warming scenario and the consequence of climate changes increasingly emphasise the requirement to consider this environmental stressor in ecotoxicological and monitoring studies, with evaluations at the lethal level but also at the sub-lethal and biochemical levels.

## Figures and Tables

**Table 1:** Cu and oxyfluorfen (OXY) concentrations (μg L^−1^ and mg L^−1^, respectively) in water samples collected in the field after depuration time, at the beginning of the bioassays and in organisms’ tissues after exposure at each condition (CTL—control; CTLS—solvent CTL). LOQ means the limit of quantification (Cu: 19.99 μg L^−1^; OXY: 10.68 μg L^−1^).

	Nominal	Measured			
			15 °C	20 °C	25 °C
		Water	Tissue	Tissue	Tissue
**Cu** **(µg L^−1^)**	Field	<LOQ	<LOQ	<LOQ	<LOQ
	Depuration	<LOQ	<LOQ	<LOQ	<LOQ
	CTL	<LOQ	<LOQ	<LOQ	<LOQ
	81.28	78.33 (±0.0018)	34.34 (±0.0017)	51.63 (±0.0008)	
	101.6	100.89 (±0.0310)	35.43 (±0.0026)	56.36 (±0.0038)	
	127.0	124.50 (±0.0230)	46.43 (±0.0028)	78.30 (±0.0163)	
	158.8	156.87 (±0.0215)	55.03 (±0.0101)	102.25 (±0.0108)	
**Oxyfluorfen** **(mg L^−1^)**	Field	<LOQ	<LOQ	<LOQ	<LOQ
	Depuration	<LOQ	<LOQ	<LOQ	<LOQ
	CTL	<LOQ	<LOQ	<LOQ	<LOQ
	CTLS	<LOQ	<LOQ	<LOQ	<LOQ
	1.88	1.92 (±0.0620)	0.69 (±0.0022)	1.00 (±0.0039)	
	2.81	2.49 (±0.0873)	0.87 (±0.0031)	1.29 (±0.0055)	
	4.22	4.49 (±0.0952)	1.62 (±0.0034)	2.30 (±0.0081)	
	6.33	6.25 (±0.1286)	2.22 (±0.0013)	3.87 (±0.0060)	
	9.49	8.16 (±0.3647)	2.83 (±0.0046)	5.10 (±0.0022)	
	14.24	14.27 (±0.2999)	5.15 (±0.0011)	7.60 (±0.0019)	

**Table 2:** Determination of LCx values (x = 10, 20 and 50) and respective lower and upper bounds, to a confidence level of 95%, which is indicated between parentheses, to Cu and oxyfluorfen on C. edule when exposed to different temperatures (15 °C and 20 °C) for 96 h. No effect concentration (NOEC) and the lowest effect concentration (LOEC) are also described.

	Cu (mg L^−1^)		Oxyfluorfen (mg L^−1^)	
	15 °C	20 °C	15 °C	20 °C
**NOEC**	0.081	-	2.813	-
**LOEC**	0.102	0.081	4.219	1.875
**LC_10_**	0.119 (0.061–0.153)	0.075 (0.002–0.116)	6.781 (0.000–6.580)	1.371 (0.00–6.580)
**LC_20_**	0.157 (0.114–0.187)	0.121 (0.068–0.155)	12.258 (7.087–20.989)	7.098 (6.856–24.041)
**LC_50_**	0.229 (0.199–0.265)	0.209 (0.176–0.248)	22.735 (16.324–48.510)	18.52 (11.238–44.868)

## Data Availability

The data presented in this study are available upon request from the corresponding authors.
